# The effect of topical ketorolac tromethamine on macular thickening after phacoemulsification in diabetic patients

**DOI:** 10.1186/s12886-023-03077-y

**Published:** 2023-07-14

**Authors:** Hossein Mohammad-Rabei, Hamideh Sabbaghi, Mehdi Emamverdi, Saeed Karimi, Alireza Ramezani, Homayoun Nikkhah, Bahareh Kheiri, Mehdi Yaseri, Kourosh Sheibani, Razieh Bahreini

**Affiliations:** 1 Ophthalmic Research Center, Research Institute for Ophthalmology and Vision Science, Shahid Beheshti University of Medical Sciences, Tehran, Iran; 2 Department of Ophthalmology, Torfe Hospital, Shahid Beheshti University of Medical Sciences, Tehran, Iran; 3 Ophthalmic Epidemiology Research Center, Research Institute for Ophthalmology and Vision Science, Shahid Beheshti University of Medical Sciences, Tehran, Iran; 4 Department of Optometry, School of Rehabilitation, Shahid Beheshti University of Medical Sciences, Opposite to Bou-Ali Hospital, Damavand Ave., Tehran, Iran; 5 School of Public Health and Public Health Research Institute, Tehran University of Medical Sciences, Tehran, Iran; 6 Basir Eye Health Research Center, Tehran, Iran

**Keywords:** Phacoemulsification, Ketorolac Tromethamine, Central Retinal Thickness, Diabetes

## Abstract

**Background:**

To determine the effect of ketorolac tromethamine 0.5% in preventing post-phacoemulsification macular thickening. This randomized clinical trial. patients randomized 1:1 to receive either topical ketorolac three times a day or a placebo.

**Methods:**

A total of 101 eyes of 101 diabetic patients who were scheduled for phacoemulsification and had normal macular contour and thickness enrolled consecutively. The topical ketorolac and placebo were prescribed on the day before surgery and continued up to 4 weeks after surgery. Patients with proliferative diabetic retinopathy, a history of intravitreal injection in less than three months, a history of macular photocoagulation in less than 6 months, and any other concomitant ocular pathologies were excluded. Central macular thickness (CMT) and best corrected visual acuity (BCVA) was recorded in the follow-ups of 6, 12, and 24 weeks after the surgery and compared with the controls.

**Results:**

49 eyes in the case group and 52 eyes in the control group were analyzed. Mean BCVA was significantly improved in both groups at all follow-ups (P < 0.001 for all). There was no statistically significant difference regarding the BCVA in different time points except week 12 (P = 0.028) among the study group. In the case and control groups, CMT was increased at all follow-ups (P < 0.05). There was no statistically significant difference when comparing the two groups regarding the mean of CMT at any time point postoperatively (P > 0.05 for all).

**Conclusion:**

Based on our findings, topical ketorolac tromethamine 0.5% is not effective in the prevention of post-phacoemulsification macular thickening in diabetic patients.

**Trail registration:**

The study protocol was registered into www.clinicaltrial.gov with the RCT registration number NCT03551808. (2018/06/11 )

**Clinical trial registration number:**

NCT03551808.

## Background

Cataract is a common cause of visual impairment in elderly individuals all around the world [1]. Nowadays, cataract surgery is the most prevalent intraocular surgery at an increasing rate from 46% to 2006 to 51.4% in 2010 in Iran [2, 3].

In spite of the recent improvements in the surgical techniques of phacoemulsification, some postoperative complications have been reported. A higher percentage of macular edema (ME) was reported in diabetic patients (12%) compared with non-diabetic individuals (6%) undergoing cataract surgery [3–7].

Although there is no agreement regarding the main pathophysiological mechanisms of postoperative ME following phacoemulsification, some studies have suggested that cataract surgery can induce inflammatory reactions in the posterior ocular segment. In a study by Xu et al., it was shown that the expression of chemokine (C-C motif) Ligand 2 and interleukin- 1β genes and protein secretion can occur in both retina and choroid after the cataract surgery [4]. According to the literature, posterior capsular rupture, dropped lens into the vitreous cavity, vitreous loss, existence of ME in the fellow eye, implantation of intraocular lens (IOL) in the anterior chamber, usage of topical prostaglandins, postoperative uveitis, particularly diabetes have been reported as the risk factors for postoperative ME following the cataract surgery [8–10].

In a prospective cohort study, a significantly higher rate of ME was observed in diabetic patients compared with non-diabetic individuals [11] and it has been shown that postoperative ME is more probable to progress in diabetic patients [12].

Ketorolac tromethamine is a potent non-steroidal anti-inflammatory drug (NSAID) that inhibits cyclooxygenase enzymes and thus decreases the level of ocular prostaglandins and consequently the chance of cystoid ME when applied topically [13–16]. In the study by Nikkhah et al., [17] the effective and sustainable influence of the topical ketorolac was reported in diabetic patients to treat diabetic ME, the same finding was also reported by Elsawy et al., [18] in spite of the above-mentioned studies, inconclusive therapeutic effects of topical NSAIDs to prevent post-phacoemulsification ME was determined by a meta-analysis conducted in 2016 [19]. The present randomized clinical trial was conducted to determine the prophylactic effect of topical ketorolac tromethamine 0.5% on post-phacoemulsification macular thickening compared to placebo in diabetic patients.

## Methods

In this randomized clinical trial (RCT), a total of 101 eyes from 101 diabetic patients who were scheduled for cataract surgery at Torfeh Medical Center, Shahid Beheshti University of Medical Sciences were included. The study protocol was registered into www.clinicaltrial.gov with the RCT registration number NCT03551808 ( 11/06/2018).

The study adhered to the tenets of the Declaration of Helsinki and was approved by the Ethics Committee of the Ophthalmic Research Center, Shahid Beheshti University of Medical Sciences. All the study procedures were explained to all patients and a signed consent form was obtained from each patient before entry to the study. We used the CONSORT reporting guidelines for our trial [20].

### Participants

Diabetic patients who were scheduled to undergo cataract surgery were included. All patients had controlled diabetes with fasting blood sugar (FBS) of 126 mg/dl or less and glycated hemoglobin A1c (HbA1c) of less than 7% at the time of study entry. Patients with glaucoma, uveitis, any previous intraocular surgery, any intravitreal injection in less than three months, macular laser photocoagulation in less than 6 months, presence of proliferative diabetic retinopathy (PDR) or any macular disease, cyclo-refraction of ≥ ± 6 diopter (D), hazy media to obtain the high-quality images of the optical coherence tomography (OCT), baseline central macular thickness (CMT) ≥ 280 μm excluded. Also, patients with any kind of intraoperative complications and those who lost to follow-up were excluded.

All the study subjects were interviewed to obtain demographic data as well as the patients’ health and ocular history by an expert technician. All patients were ordered to perform the laboratory tests including FBS, creatinine, urea, and HbA1c at the time of study recruitment.

Participants were randomized using the permuted block randomization method in two groups. In multiple studies and a meta-analysis, it was shown that starting NSAID eye drops are more effective in decreasing inflammation and ME, if started 1 to 3 days prior to cataract surgery [12, 21]. Cases were instructed to use ketorolac tromethamine 0.5% (Sinarolac®; Sina Darou, Tehran, Iran) eye drop three times daily one day before cataract surgery and continued it up to four weeks after the surgery, while the control group only received a placebo treatment (the preservative-free artificial tears (Sinalone®; Sina Darou, Tehran, Iran)) which put into the drug container as same as the ketorolac eye drop). Postoperatively, betamethasone 0.1% (Betasonate®; Sina Darou, Tehran, Iran) eye drop was prescribed to all study subjects in both groups 4 times per day in the first week and it was gradually tapered to once a day in the fourth week. Also, chloramphenicol 0.5% (Cholobiotic®; Sina Darou, Tehran, Iran) eye drop was applied 4 times per day for one week after the surgery.

Comprehensive visual and ocular examinations were performed on all study subjects. Refractive error measurement was performed either by auto-refractometer (RM-8800; Topcon Medical, Oakland, NJ, USA) or retinoscope (HEINE BETA®200; Germany). BCVA was assessed using a Snellen E-chart at a distance of 6 m by a trained optometrist. In addition, a biomicroscopic examination was performed to evaluate the anterior segment. Intraocular pressure was measured using the applanation tonometer (Goldmann applanation tonometer, Haag-Streit, USA). Fundus examination was conducted using an indirect ophthalmoscope through dilated pupil by a 78D lens to determine the stage of diabetic retinopathy according to international classification [22].

Spectral-domain OCT (SD-OCT, Heidelberg Engineering OCT Spectralis, USA) was performed for all study subjects to measure the CMT. IOL power calculation was conducted using A-scan (ZEISS IOL Master 500; Germany) at the baseline examination. All ophthalmic examinations and cataract surgery were performed by an expert anterior segment ophthalmologist (HMR).

### Surgical technique

The procedure was performed under topical or general anesthesia, according to the patient’s condition, by a single anterior segment surgeon (HMR). After a 2.8 mm clear corneal incision, phacoemulsification was performed using the divide and conquer technique, and the IOL was inserted in the capsular bag using the Monarch II injector and a C cartridge (Alcon Laboratories Inc., Fort Worth, TX, USA). After irrigation and aspiration, the anterior chamber was formed with the balanced salt solution. There was no wound leakage. Subconjunctival betamethasone (4 mg) and ceftazidime (100 mg) were injected, and the eyes were patched.

### Follow-up examinations

All patients were routinely examined the next day, one week, and one month after the surgery for possible surgical complications. Follow-up examinations including OCT imaging and BCVA measurement were conducted at the three-time points of 6, 12, and 24 weeks following the surgery for both cases and controls.

### Randomization

All participants were randomly assigned into study groups of cases and controls using the permuted block randomization method with a random block length of 2, 4, 6, and 8. The randomization list was generated by a computer-based program and the details of the random sequence were concealed from researchers. Whenever a new patient entered the study a new envelope (which contained the group for the order sequence of the patient) was opened and the group was revealed. Participant enrollment and intervention assignment were carried out (HM, HE, and AR).

### Blinding

Evaluation of outcomes was performed by a researcher who was uninformed about the group assignment. The study protocol and the type of treatment were concealed from our participants.

### Sample size

To have a power of 90% to detect a 30 μm difference of CMT in 12 weeks, we needed 50 subjects in each group. It was based on the assumed standard deviation of 0.26 which was obtained in the pilot phase of this study. The probable attrition of up to 30% and type one error of 0.05 were also considered. Fifty-eight diabetic patients having the eligibility criteria were enrolled in each group due to overcome the possible loss of follow-up.

### Outcome measures

The changes of CMT and BCVA at the three-time points of weeks 6, 12, and 24 compared to the baseline were considered as the primary outcome measures.

### Statistical analysis

The normal distribution of quantitative data was assessed by the Shapiro-Wilks test and Q-Q plot. To describe data, we used, mean and standard deviation, median and range, frequency and percentage. To compare the baseline variables between the two groups we used an independent t-test, Mann-Whitney test, chi-square test, and Fisher exact test. Evaluation of the variables’ changes in different follow-ups was performed using a linear mixed model. To compare the groups in different follow-ups we used Multivariate Analysis of Variance (MANOVA). All statistical analysis was performed using SPSS software Version 25 (IBM Corp. Armonk, NY). All tests were two-sided and P-value less than 0.05 was considered statistically significant.

## Results

**Fig. 1:**
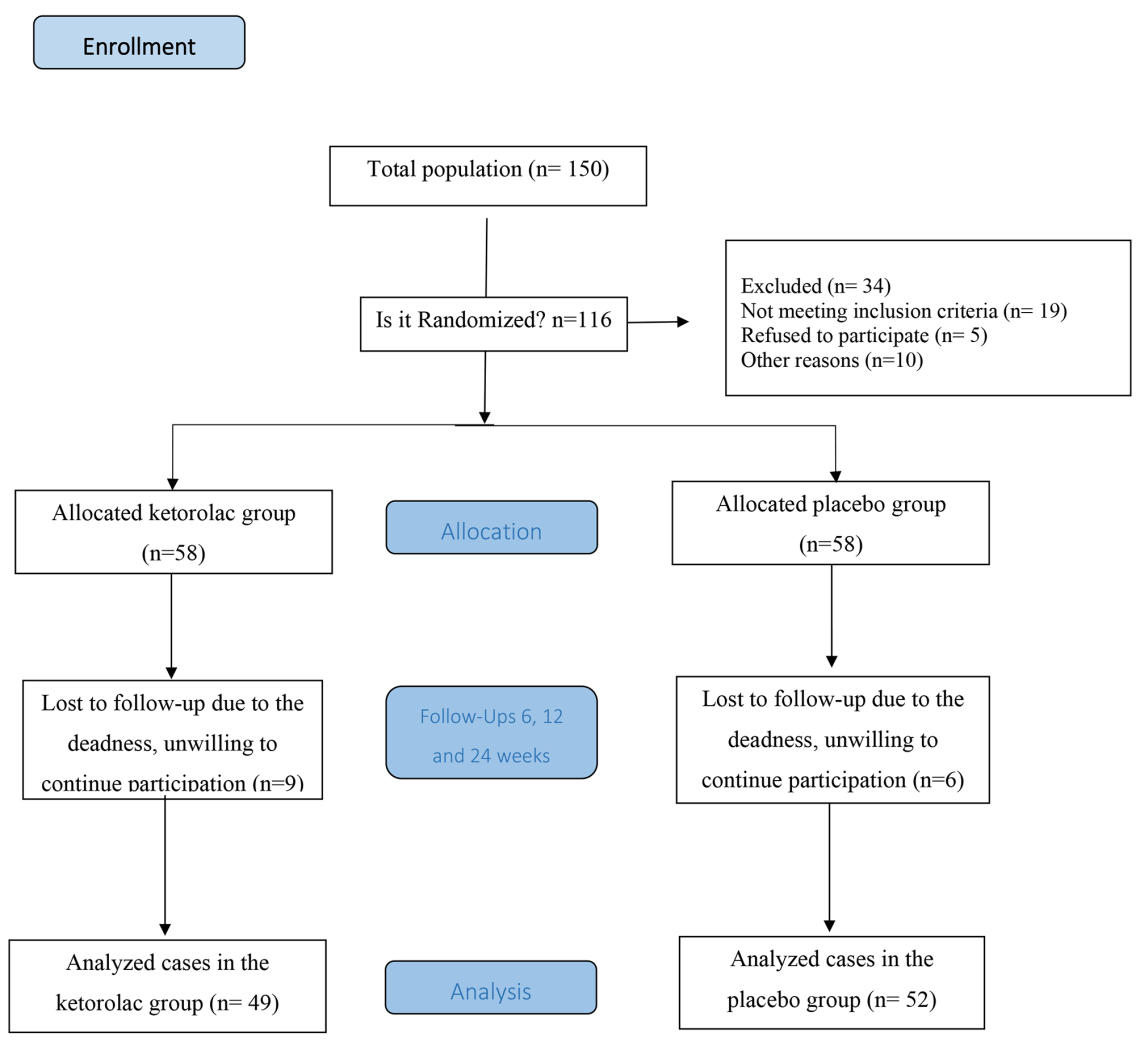
The flowchart of the present study

This study enrolled 116 eligible patients, but the data of 15 patients were not included in the statistical analysis due to miss follow-up Finally, a total of 101 eyes out of 101 diabetic patients (mean age of 64 ± 10 and range of 45 to 95 years) were included (Fig. 1).

Table 1 presents the baseline characteristics of both case and control groups. As shown, there was no statistically significant difference between the study groups regarding age, sex, the spherical equivalent of refractive error, duration of diabetes, diabetic medications, stages of diabetic retinopathy, and laboratory findings. As shown in Table 2, the mean CMT at baseline was (Case: 253 ± 24 μm and Control: 260 ± 20 μm) statistically significantly increased in both groups after the surgery in week 6 (Case: 282 ± 56 μm and Control: 312 ± 94 μm) and week 12 (Case: 279 ± 39 and Control: 292 ± 53 μm). However In week 24 (case 284 ± 71 μm, control 280 ± 41 μm) increase in CMT was greater in the control group compared with the case group in the follow-ups of 6 and 12 weeks, but the mean CMT changes from the baseline were not significantly different among the study groups. As presented in Table 3, the mean BCVA was significantly improved in both groups in all three-time points of 6, 12, and 24 weeks compared to the baseline examination (P < 0.05 for all comparisons), while the changes of BCVA were not significantly different in weeks 6 and 24 when comparing the two groups. However, the cases group had a significantly better BCVA compared with controls at week 12 postoperatively (0.07 ± 0.07 in cases vs. 0.2 ± 0.26 LogMAR in controls, P = 0.028), however, no significant difference was found in the other time points.

**Table 1:** Baseline characteristics in the case and the control groups

Parameter	Level	Total	Groups		P
			Case (n = 49)	Control (n = 52)	
Age (yrs)	Mean ± SD	64 ± 10	65 ± 9	64 ± 10	0.290†
	Median (range)	63 (45 to 95)	64 (45 to 87)	63 (45 to 95)	
Sex	Male	49 (48.5%)	23 (46.9%)	26 (50.0%)	0.758*
	Female	52 (51.5%)	26 (53.1%)	26 (50.0%)	
Pre SE (D)	Mean ± SD	-0.81 ± 6.9	-2.06 ± 3.2	0.35 ± 9	0.22†
	Median (range)	-1.5 (-11 to 43.5)	-1.75 (-11 to 2.75)	-1.31 (-4.5 to 43.5)	
Duration of Diabetes (month)	Mean ± SD	102 ± 83	93 ± 80	110 ± 86	0.289‡
	Median (range)	84 (1 to 324)	60 (1 to 276)	96 (1 to 324)	
Medications Diabetes	Oral Medication	66 (64.8%)	28 (68.3%)	27 (0.0%)	0.197**
	Insulin Therapy	18 (17.6%)	8 (19.5%)	4 (8.3%)	
	Diet	18 (17.6%)	5 (12.2%)	13 (27.1%)	
Stages of DR	NO Sign	66 (64.7%)	36 (73.5%)	30 (56.6%)	0.09**
	Mild NPDR	21 (20.6%)	10 (20.4%)	11 (20.8%)	
	Moderate NPDR	6 (5.9%)	1 (2.0%)	5 (9.4%)	
	Severe NPDR	9 (8.8%)	2 (4.1%)	7 (13.2%)	
FBS (mg/dl)	Mean ± SD	114 ± 8	113 ± 10	115 ± 7	0.362‡
	Median (range)	118. (71 to 124)	118 (71 to 124)	118 (98 to 124)	
HbA1C (mmol/mol)	Mean ± SD	6.51 ± 0.4	6. 61 ± 0.42	6.41 ± 0.46	0.733‡
	Median (range)	6.5 (5.3 to 7)	6.8 (6.00 to 7)	6.4 (5.3 to 7)	
Cr (mg/dl)	Mean ± SD	1.44 ± 3.3	1.05 ± 0.18	1.86 ± 4.73	0.576‡
	Median (range)	1 (0.1 to 29)	1 (0.8 to 1.6)	1.05 (0.1 to 29)	
Urea (mg/dl)	Mean ± SD	35.6 ± 12.9	34 ± 10	37.2 ± 15.3	0.522‡
	Median (range)	33 (1.9 to 80)	33 (19 to 61)	35 (1.9 to 80)	

SE, spherical equivalent; Cr, creatinine; FBS, fast blood sugar; HbA1C, hemoglobin A1C; NPDR, non- proliferative diabetic retinopathy; DR, diabetic retinopathy; P, probability; SD, standard deviation; w, week; D, diopter; yrs, years

† Based on t-test

‡ Based on Mann-Whitney test

* Based on Chi-Square tesst

** Based on Fisher exact test

**Table 2:** The mean and changes of CMT in case and control groups in different follow ups

CMT (µm)	Total (n = 101)		Case (n = 49)		Control (n = 52)		Diff	95% CI		P
	Mean ± SD	Median (range)	Mean ± SD	Median (range)	Mean ± SD	Median (range)		Lower	Upper	
Pre	257 ± 22	258 (183 to 296)	253 ± 24	253 (183 to 294)	260 ± 20	262 (211 to 296)	-6.82	-15.75	2.10	0.11
6 W	299 ± 80	278 (172 to 602)	282 ± 56	271 (172 to 487)	312 ± 94	286 (217 to 602)	-30.08	-74.84	14.69	0.556
12 W	287 ± 48	284 (172 to 453)	279 ± 39	282 (172 to 338)	292 ± 53	285 (209 to 453)	-12.50	-41.62	16.62	0.131
24 W	282 ± 56	272 (170 to 499)	284 ± 71	270 (170 to 499)	280 ± 41	273 (213 to 409)	3.93	-37.38	45.23	0.945
Pre-6 W	34 ± 81	12 (-85 to 355)	22 ± 44	9 (-11 to 196)	44 ± 101	12 (-85 to 355)	-21.40	-68.83	26.02	0.58
P-Whitin§			0.006		0.034					
Pre-12 W	18 ± 28	15 (-46 to 126)	12 ± 18	11 (-15 to 49)	22 ± 34	17 (-46 to 126)	-9.57	-26.80	7.65	0.322
P-Whitin§			< 0.001		0.018					
Pre-24 W	19 ± 50	7 (-13 to 223)	23 ± 58	12 (-13 to 223)	16 ± 44	3 (-12 to 180)	7.61	-29.94	45.15	0.908
P-Whitin§			0.089		0.108					

CMT, central macular thickness; SD, standard deviation; w, weeks; Diff, difference; CI, confidence interval P, probability

§Based on t-test

† Based on generalized linear mixed model (GLM)

**Table 3:** The mean and changes of BCVA (LogMAR) in case and control groups in different follow ups

BCVA (LogMAR)	Total (n = 101)		Case (n = 49)		Control (n = 52)		Diff	95% CI		P
	Mean ± SD	Median (range)	Mean ± SD	Median (range)	Mean ± SD	Median (range)		Lower	Upper	
Pre	0.48 ± 0.31	0.4 (0 to 1.3)	0.52 ± 0.33	0.4 (0.05 to 1.3)	0.45 ± 0.28	0.4 (0 to 1.3)	0.07	-0.05	0.18	0.88
6 W	0.14 ± 0.23	0.08 (0 to 1.3)	0.07 ± 0.1	0.05 (0 to 0.4)	0.21 ± 0.29	0.1 (0 to 1.3)	-0.13	-0.26	-0.01	0.198
12 W	0.14 ± 0.21	0.1 (0 to 1)	0.07 ± 0.07	0.05 (0 to 0.22)	0.2 ± 0.26	0.13 (0 to 1)	-0.13	-0.25	-0.01	0.028
24 W	0.12 ± 0.19	0.05 (0 to 0.7)	0.14 ± 0.18	0.1 (0 to 0.7)	0.1 ± 0.2	0 (0 to 0.7)	0.04	-0.11	0.20	0.723
Pre-6 W	-0.32 ± 0.32	-0.3 (-1 to 0.6)	-0.42 ± 0.26	-0.4 (-1 to 0)	-0.23 ± 0.34	-0.14 (-0.9 to 0.6)	-0.19	-0.36	-0.02	0.295
P-Whitin§			< 0.001		< 0.001					
Pre-12 W	-0.32 ± 0.28	-0.3 (-1 to 0.48)	-0.37 ± 0.25	-0.37 (-1 to 0.05)	-0.27 ± 0.3	-0.28 (-0.9 to 0.48)	-0.10	-0.27	0.07	0.655
P-Whitin§			< 0.001		< 0.001					
Pre-24 W	-0.33 ± 0.23	-0.3 (-0.9 to 0)	-0.34 ± 0.21	-0.34 (-0.78 to 0)	-0.31 ± 0.27	-0.26 (-0.9 to 0)	-0.03	-0.22	0.16	0.152
P-Whitin§			< 0.001		0.004					

BCVA, best corrected visual acuity; LogMAR, logarithm of the minimum angle of resolution; SD, standard deviation; w, weeks; Diff, difference; CI, confidence interval; P, probability

§Based on t-test

† Based on generalized linear mixed model (GLM)

## Discussion

In the current Randomized Clinical Trial (RCT), we investigated the prophylactic effect of ketorolac eye drop on post-phacoemulsification macular thickening in diabetic patients. We found that prophylactic ketorolac tromethamine 0.5% initiated the day before the surgery and continued up to 4 weeks in addition to topical corticosteroid was not associated with better visual outcome or lower macular thickness after the cataract surgery, compared to placebo except for follow- up in week 12, which was in favor of the ketorolac group. As well, the mean change in CMT was similar between the two study groups.

There are several studies in the literature investigating the prophylactic or therapeutic effects of NSAIDs on ME following cataract surgery. Sivaprasa et al. evaluated the effects of NSAIDs on the treatment of acute and chronic ME following cataract surgery in a systematic review that included seven RCTs [8]. Three trials examined the effect of topical NSAIDs on acute ME. No positive evidence was found regarding the effects of NSAIDs on acute ME after the cataract surgery. Their results were similar to our study, however, they administrated NSAIDs after the occurrence of ME, not as a prophylaxis.

Ticly et al. [16] conducted an RCT to evaluate the effects of prophylactic ketorolac on post-phacoemulsification ME. Eighty-one patients were randomized into ketorolac and placebo groups. The ketorolac was administrated 4 times a day, started 3 days before the surgery, and continued up to 5 weeks postoperatively. The authors concluded no difference between prophylactic ketorolac and placebo on the rate of post-phacoemulsification ME, which was in concordance with the current study. Lim et al. [23] reviewed the prophylactic effect of NSAIDs on the prevention of ME after cataract surgery. Nine trials compared topical NSAIDs in addition to topical corticosteroids versus corticosteroids alone. Six studies reported central retinal thickness at the end of the follow-up period, while three studies reported a change in thickness from baseline. The results were not consistent (I^2^ = 87%). Some studies were in favor of NSAIDs plus steroids and somewhere in favor of steroids alone.

Furthermore, the prophylactic effect of topical ketorolac against the development of post-cataract surgery ME in diabetic patients was evaluated by Elsawy et al. [24]. The patients were divided into ketorolac group, who received topical ketorolac tromethamine 0.4% twice a day for 12 weeks in addition to dexamethasone 0.1% four times a day and control group, who received only topical dexamethasone 0.1%. Contrary to the present study, the above-mentioned trial found a significantly increased rate of postoperative ME in diabetic patients in the control group compared to the ketorolac group. Singal et al. [6] evaluated topical nepafenac in the prevention of post-phacoemulsification ME in patients with diabetic retinopathy. In an RCT, the patients were assigned into two groups to receive either nepafenac in addition to topical corticosteroid or corticosteroid and placebo. Topical nepafenac was started 3 times daily the day before the surgery and continued for 90 days after the surgery. The authors found that significantly fewer patients developed ME in the nepafenac group (P < 0.001). The mean central subfield thickness was also significantly less in the nepafenac group (P ≤ 0.005). These results were not in agreement with our study. In the present study, we did not find any beneficial effect of topical ketorolac to induce greater improvement in visual acuity and reduce macular thickness after cataract surgery in diabetic patients.

The risk factors for the increase of macular thickness after phacoemulsification in diabetic patients are not well defined. However, some factors are diagnosed to increase the risk of macular thickening such as the presence of insulin-dependent diabetes mellitus, duration of diabetes ≥ 10 years, the severity of diabetic retinopathy, and prior treated diabetic ME. The results of the present study are in contrast to the findings of Elsawy et al., [24] and Singal et al. [6]. Our patients (both ketorolac and placebo groups) had good diabetes control (FBS ≤ 126 mg/dl and HbA1c < 7%), most of them had no diabetic retinopathy or mild non-proliferative diabetic retinopathy and used oral agents or just were on diet to control their diabetes. Furthermore, the mean duration of diabetes was less than 10 years in both groups. Taking all these factors into consideration, one can assume that our patients were minimally affected by diabetes. That’s probably why we did not find any differences between the two groups in terms of visual acuity improvement and macular thickness changes following the cataract surgery.

Our study has significant limitations. A low sample size in each group may influence the statistical significance of the results. Also, we did not subgroup analysis based on diabetic retinopathy stages and normal eyes. Also maybe some confounding factors such as surgery time, cataract severity, and post-operation inflammation level influence our results and also the lack of fluorescein angiography imaging was a possible limitation of the current study.

## Conclusions

In conclusion, the current randomized clinical trial suggests that prophylactic topical ketorolac 0.5% three times a day might not decrease CMT and improve visual acuity compared to placebo following cataract surgery in patients with diabetes. However, further studies with larger sample sizes are recommended.

## Data Availability

The datasets used and/or analyzed during the current study are available from the corresponding author upon reasonable request.

## List of Abbreviations

CMT: Central macular thickness
ME: Macular edema
NSAID: Non-steroidal anti-inflammatory drug
FBS: Fasting blood sugar
HbA1c: Glycated hemoglobin A1c
PDR: Proliferative diabetic retinopathy
OCT: Optical coherence tomography
RCT: Randomized Clinical Trial
